# Occupational burnout and empathy influence blood pressure control in primary care physicians

**DOI:** 10.1186/s12875-017-0634-0

**Published:** 2017-05-12

**Authors:** Oriol Yuguero, Josep Ramon Marsal, Montserrat Esquerda, Jorge Soler-González

**Affiliations:** 1 Institut Universitari d’Investigació en Atenció Primària Jordi Gol (IDIAP Jordi Gol), Barcelona, Spain; 2 Regió Sanitària de Lleida. Institut Català de la Salut, Lleida, Spain; 3 Unitat de Suport a la Recerca Lleida. Institut Universitari d’Investigació en Atenció Primària Jordi Gol, Barcelona, Spain; 4 Institut Borja Bioètica, Barcelona, Spain

**Keywords:** Hypertension, Primary Care, Bioethics, Doctor-Patient Relation, Faculty Development

## Abstract

**Background:**

Good physician-patient communication can favor the adoption of healthy lifestyle habits, which is essential in high blood pressure (BP) management. More empathic physicians tend to have lower burnout and better communication skills. We analyzed the association between burnout and empathy among primary care physicians and nurses and investigated the influence on BP control performance.

**Methods:**

Descriptive study conducted in 2014 investigating burnout and empathy levels in 267 primary care physicians and nurses and BP control data for 301,657 patients under their care. We administered the Maslach Burnout Inventory and the Jefferson Scale of Physician Empathy and defined good BP control as a systolic BP <130 mmHg.

**Results:**

Low burnout and high empathy were observed in 58.8% and 33.7% of practitioners, respectively. Burnout and empathy were significantly negatively associated (p < 0.009). Practitioners with high empathy and low burnout had significantly better BP control and performance than those with low empathy and high burnout (p < 0.05).

**Conclusions:**

Low burnout and high empathy were significantly associated with improved BP control and performance, possibly in relation to better physician/nurse-patient communication.

## Background

The doctor-patient relationship is a key part of clinical practice and has experienced many changes throughout the history of medicine, [1] particularly in recent decades, in response to shifting roles, responsibilities, and expectations brought about by social changes, scientific and technological advances, and the growing prominence of the information society.

Occupational burnout has gone from being a problem restricted to emergency medicine to one that affects the whole health care system, [2] right down to primary care [3].

Although the concept of burnout was introduced by Freudenberger as early as 1974, [4] it was Maslach who designed what is now the most widely used instrument for measuring burnout: the Maslach Burnout Inventory (MBI). [5] Burnout symptoms are similar to those seen in chronic stress, and can be psychosomatic, behavioral, emotional, and/or defensive [6, 7]. One strategy for reducing the effects of burnout is to promote understanding and empathy among care providers, [8, 9] and this would have an obvious knock-on effect in terms of improved clinician-patient communication. Low empathy appears to be linked to high burnout, and particularly to certain components of the syndrome [10]. A recent study by our group found a significant association between empathy and burnout among family doctors in our health district, although we found no evidence that either of these factors had an impact on sick leave prescribing habits [11].

An empathic health care professional displays a caring attitude and is capable not only of understanding his or her patient’s experiences and feelings but also of communicating this understanding [12]. According to Neumann, [13] empathy displayed during a doctor-patient visit could have numerous beneficial effects that would result in tangible improvements in clinical outcomes.

In a study published in 2011, Hojat et al. [14]. reported preliminary findings that supported a positive association between physician empathy and clinical outcomes in a group of diabetic patients. This was the first study of its kind to provide evidence that physician empathy could have immediate clinical benefits and should be considered a key competence. Hojat et al. did not detect any other clinical changes, but we believe that it would be interesting to continue this line of research by investigating similar links in other diseases that affect a large proportion of the population, such as dyslipidemia, high blood pressure, or heart failure. We chose to investigate the association between empathy and burnout levels in primary care physicians and nurses and BP control and performance, as lifestyle changes are the first step in the treatment of high BP, particularly in the early stages of disease, which are the most prevalent. Prompted thus by the work of Hojat et al. and our recent observation of an association between empathy and burnout among family physicians, we investigated whether these factors might influence blood pressure (BP) control performance in primary care.

Hypertension is a highly prevalent condition in the general population and is frequently managed by primary care providers. In Spain alone, over 10 million adults (approximately 35% of the adult population) and around 68% of adults aged over 60 years have high BP [15].

While extensive guidelines exist on the management of hypertension, and a considerable proportion of the Spanish hypertensive population have been identified and are receiving pharmacologic therapy, levels of control are poor, particularly in the case of systolic BP [16]. At the primary care level, family physicians and nurses are often involved in diagnosing hypertension, prescribing initial treatment (lifestyle modifications and/or pharmacologic therapy), monitoring treatment adherence, and performing regular checks.

Several recent studies have analyzed treatment adherence and BP control performance in primary care [17]. Poor control has been associated with multiple factors, including the use of minimally active drugs, reluctance among physicians to combine antihypertensives with other medications, [18] poor adherence to treatment, [19] and failure to implement lifestyle modifications, such as dietary changes.

In this study we tested the hypothesis that patients under the care of family physicians and nurses with low burnout and high empathy would have better BP control and management than those under the care of physicians and nurses with high burnout and low empathy.

## Methods

### Participants

We e-mailed all primary care physicians and nurses working in the health care district of Lleida, inviting them to participate in this study. Lleida is a Catalan province with a population of approximately 400,000 and about 35% live in the capital city [20]. Of the 435 primary care providers contacted, 267 agreed to participate in this study. This corresponds to a response rate of 61.3%, which is higher than the target of 50% established in a previous study by our group in which we assessed levels of empathy among primary care providers in the same district [21]. We calculated that to detect an increase or decrease of 0.5 mmHg in systolic BP, we would need 19,607 patients per group.

In Catalonia, primary care health centers are open 12 h a day from Monday to Friday and providers work 37.5 h a week. Nights and weekends are covered by emergency centers.

All the survey respondents work in the public primary care service, which covers over 80% of primary care provision in Catalonia. For our analyses, we distinguished between physicians and nurses.

### Instruments

Burnout was measured using the Spanish version of the MBI, which consists of 22 items that evaluate feelings of emotional exhaustion, depersonalization, and (reduced) personal accomplishment. Each item is scored using a 7-point Likert frequency scale ranging from “never” (0) to “every day” (6). The Spanish version was validated by Moreno-Jimenez [22]. The physicians and nurses were divided into three groups (low, medium, and high burnout) according to scores for each of the dimensions and for the scale as a whole. The cutoff points used for the three categories are those indicated by the authors of the questionnaire. The MBI is widely recognized and has been administered to physicians and nurses both inside and outside Spain in numerous studies [23–25].

Empathy was measured using the validated Spanish version of the Jefferson Scale of Physician Empathy (JSPE), [21] which is a 20-item scale that measures physician empathic orientation and behavior. It is also scored using a 7-point Likert system, ranging from strongly disagree (1) to strongly agree (7).

We also divided the physicians and nurses into three groups (low, medium, and high) according to their level of empathy. In this case, however, there were no pre-established cutoffs. The average empathy score is considered to lie around 125 points and we followed previous strategies of classifying empathy levels as high for mean scores plus 2 SDs and as low for mean scores minus 2 SDs.

BP data were obtained for the patients under the care of the 267 physicians and nurses who participated in the study from the centralized primary health care computer system. Data for 301,657 patients were analyzed for the period January 2013 to June 2013 and a distinction was made between patients with adequate and inadequate BP management. Management was considered to be adequate when at least two correctly recorded BP measurements, taken on different days, were included in the patients’ records for the period studied. All the primary care centers have the same tensiometer and are periodically revised to make the same records and to ensure quality of measurements, and usually placed in the same way to ensure similar measurements.

Patients were classified as hypertensive if their records included mention of the hypertension code or if they had at least two systolic BP readings of >140 mmHg or a diastolic BP reading of >90 mmHg. In accordance with the recently published criteria of the European Society of Hypertension, [26] patients with a systolic BP of <130 mmHg were considered to have good BP control. We also recorded how many times each patient had their BP measured during the study period, regardless of whether or not they were hypertensive.

### Other variables

The following data were collected for each physician and nurse: age, sex, and geographic area. The health care centers were categorized as urban (those in the capital city) or rural (those in other towns and villages).

### Study design and data analysis

Study data were collected from the questionnaires completed by the health care providers and from the centralized primary care data system. Numerical data were described using means and standard deviation or medians and interquartile ranges, while categorical and ordinal data were described using absolute and relative frequencies. We also calculated 95% confidence intervals for descriptive data. MBI and JSPE scores were each grouped into three categories—low, moderate, and high—and systolic BP was classified as <130 mmHg (good control), 130–140 mmHg (normal high BP), and >140 mmHg (poor control). Associations between BP control and management and levels of empathy and burnout were described separately for physicians and nurses using the Chi-Square linear-by-linear test for ordinal variables and analysis of variance for numerical data. For all analyses, a p-value of < 0.05 was considered statistically significant. SPSS Software was used for data management and statistical analysis.

## Results

In total, 267 primary care physicians and nurses (58 men and 209 women) completed the MBI and JSPE, and BP data were collected for 301,657 patients under their care. The study covered 22 health centers, 6 of which were classified as urban. The sociodemographic characteristics of the providers are shown in Table 1, together with a summary of the percentages of providers with low, high, and medium burnout and empathy. We found no differences in burnout and empathy percentages for men and women (data not shown). The make-up of the sample reflects the true study population, as there is a predominance of female primary care providers in our health district. In the year of the study, for example, 92% of family nurses and 58% of family physicians were women [27].

**Table 1:** Sociodemographic characteristics of 267 family nurses and physicians and burnout and empathy results

Sociodemographic characteristics	
Sex	
Male	58 (21.7%)
Female	209 (178.3%)
Profession	
Nurse	131 (49.1%)
Physician	126 (50.9%)
Place of Work	
Urban	111 (41.6%)
Rural	156 (58.4%)
Mean age (years)	48.1
Burnout^a^	
Low	157 (58.8%)
Medium	100 (37.5%)
High	10 (3.7%)
Empathy^b^	
Low	89 (33.3%)
Medium	88 (33%)
High	90 (33.7%)

^a^Assessed using the Maslach Burnout Inventory

^b^Assessed using the Jefferson Scale of Physician Empathy

According to the results of the MBI, 157 family physicians and nurses (58.8%) had low burnout, 100 (37.5%) had moderate burnout, and 10 (3.7%) had high burnout. These proportions were maintained for the three scales that make up the MBI: emotional exhaustion, depersonalization, and personal accomplishment. Based on the JSPE scores, 89 participants (33.3%) had low empathy, 99 (33%) had moderate empathy, and 90 (33.7%) had high empathy. Higher empathy scores were significantly associated with lower burnout scores (p < 0.05).

### Burnout/empathy of physicians and BP management and control

Table 2 and 3 show the BP results for the overall group of patients and for hypertensive patients according to whether they were under the care of physicians with low, moderate, or high empathy and burnout. Mean systolic BP was significantly lower in patients under the care of physicians with low burnout for both the overall population (p = 0.001) and patients with hypertension (p = 0.011).

**Table 2:** Blood Pressure control and results of Physician’s Empathy

Physicians		Low Empathy	Moderate Empathy	High Empathy	All	p
Patients under physicians’ care	n	42138	45070	61624	148832	
Patients with adequate blood pressure management^a^	n (%)	16691 (39.61%)	18592 (41.25%)	24166 (39.22%)	59449 (39.94%)	0.048^b^
	95% CI	[39.14% – 40.08%]	[40.8% – 41.71%]	[38.83% – 39.6%]	[39.69% – 40.19%]	
Systolic blood pressure in overall population	mean (SD)	127.79 (15.2)	127.94 (15.1)	127.5 (15.3)	127.72 (15.2)	0.009^b^
	95% CI	[127.6 – 128]	[127.7 – 128.2]	[127.3 – 127.7]	[127.6 – 127.8]	
	median [IQR]	129 [119 – 137]	129 [119 – 137]	128 [118 – 137]	129 [119 – 137]	
Blood control levels in overall population						
< 130 mmHg	n (%)	8522 (51.06%)	9457 (50.87%)	12623 (52.23%)	30602 (51.48%)	0.012^b^
	95% CI	[50.3% – 51.82%]	[50.15% – 51.58%]	[51.6% – 52.86%]	[51.07% – 51.88%]	
130 – 140 mmHg	n (%)	4992 (29.91%)	5529 (29.74%)	7086 (29.32%)	17607 (29.62%)	
	95% CI	[29.21% – 30.6%]	[29.08% – 30.4%]	[28.75% – 29.9%]	[29.25% – 29.98%]	
> 140 mmHg	n (%)	3177 (19.03%)	3606 (19.4%)	4457 (18.44%)	11240 (18.91%)	
	95% CI	[18.44% – 19.63%]	[18.83% – 19.96%]	[17.95% – 18.93%]	[18.59% – 19.22%]	
Hypertensive patients	n (%)	1242 (2.95%)	1746 (3.87%)	1654 (2.68%)	4642 (3.12%)	<0.001^b^
	95% CI	[2.79% – 3.11%]	[3.7% – 4.05%]	[2.56% – 2.81%]	[3.03% – 3.21%]	
Systolic blood pressure in hypertensive population	mean (SD)	131.05 (15.3)	130.5 (14.9)	130.58 (15.8)	130.67 (15.3)	0.703^c^
	95% CI	[130–132.1]	[129.7 – 131.3]	[129.7 – 131.5]	[130.1 – 131.2]	
	median [IQR]	130 [121–139]	130 [120–138]	130 [120–139]	130 [120–139]	
Blood control levels in hypertensive population						
< 130 mmHg	n (%)	345 (40.97%)	532 (42.19%)	518 (44.2%)	1395 (42.6%)	0.353^b^
	95% CI	[37.65% – 44.3%]	[39.46% – 44.91%]	[41.35% – 47.04%]	[40.9% – 44.29%]	
130 – 140 mmHg	n (%)	289 (34.32%)	446 (35.37%)	368 (31.4%)	1103 (33.68%)	
	95% CI	[31.12% – 37.53%]	[32.73% – 38.01%]	[28.74% – 34.06%]	[32.06% – 35.3%]	
> 140 mmHg	n (%)	208 (24.7%)	283 (22.44%)	286 (24.4%)	777 (23.73%)	
	95% CI	[21.79% – 27.62%]	[20.14% – 24.75%]	[21.94% – 26.86%]	[22.27% – 25.18%]	

^a^At least two correctly recorded readings taken on different days

^b^Chi-square linear-by-linear test

^c^Analysis of variance

**Table 3:** Blood Pressure control and results of Physician’s Burnout

Physicians		Low Burnout	Medium Burnout	High Burnout	All	
Patients under physicians’ care	n	81430	57742	9660	148832	
Patients with adequate blood pressure management^a^	n (%)	32612 (40.05%)	22968 (39.78%)	3869 (40.05%)	59449 (39.94%)	0.517^1^
	95% CI	[39.71% – 40.39%]	[39.38% – 40.18%]	[39.07% – 41.03%]	[39.69% – 40.19%]	
Systolic blood pressure in overall population	mean (SD)	127.34 (15)	128.2 (15.5)	128.1 (15.4)	127.72 (15.2)	<0.001^2^
	95% CI	[127.2 – 127.5]	[128–128.4]	[127.6 – 128.6]	[127.6 – 127.8]	
	median [IQR]	128 [118–137]	129 [119–138]	129 [119–138]	129 [119–137]	
Blood control levels in overall population						<0.001^1^
<130 mmHg	n (%)	17125 (52.51%)	11513 (50.13%)	1964 (50.76%)	30602 (51.48%)	
	95% CI	[51.97% – 53.05%]	[49.48% – 50.77%]	[49.19% – 52.34%]	[51.07% – 51.88%]	
130 – 140 mmHg	n (%)	9594 (29.42%)	6886 (29.98%)	1127 (29.13%)	17607 (29.62%)	
	95% CI	[28.92% – 29.91%]	[29.39% – 30.57%]	[27.7% – 30.56%]	[29.25% – 29.98%]	
>140 mmHg	n (%)	5893 (18.07%)	4569 (19.89%)	778 (20.11%)	11240 (18.91%)	
	95% CI	[17.65% – 18.49%]	[19.38% – 20.41%]	[18.85% – 21.37%]	[18.59% – 19.22%]	
Hypertensive Patients	n (%)	2510 (3.08%)	1683 (2.91%)	449 (4.65%)	4642 (3.12%)	<0.001^1^
	95% CI	[2.96% – 3.2%]	[2.78% – 3.05%]	[4.23% – 5.07%]	[3.03% – 3.21%]	
Systolic blood pressure in hypertensive population	mean (SD)	129.98 (14.7)	131.7 (16.1)	130.83 (15.4)	130.67 (15.3)	0.011^2^
	95% CI	[129.3 – 130.7]	[130.8 – 132.6]	[129–132.6]	[130.1 – 131.2]	
	median [IQR]	130 [120–139]	131 [121–140]	130 [123–138]	130 [120–139]	
Blood control levels in hypertensive population						0.277^1^
<130 mmHg	n (%)	802 (44.14%)	466 (39.76%)	127 (44.41%)	1395 (42.6%)	
	95% CI	[41.86% – 46.42%]	[36.96% - 42.56%]	[38.65% – 50.16%]	[40.9% – 44.29%]	
130 – 140 mmHg	n (%)	601 (33.08%)	403 (34.39%)	99 (34.62%)	1103 (33.68%)	
	95% CI	[30.91% – 35.24%]	[31.67% – 37.11%]	[29.1% – 40.13%]	[32.06% – 35.3%]	
>140 mmHg	n (%)	414 (22.78%)	303 (25.85%)	60 (20.98%)	777 (23.73%)	
	95% CI	[20.86% – 24.71%]	[23.35% – 28.36%]	[16.26% – 25.7%]	[22.27% – 25.18%]	

^a^ At least two correctly recorded readings taken on different days. ^1^: Chi-square linear-by-linear association Test ^2^: ANOVA test

Physicians with low levels of empathy had a significantly higher proportion of patients with adequate BP management (p = 0.048). However, mean systolic BP was lower among patients under the care of highly empathic physicians (p = 0.009), who also had a lower proportion of patients with hypertension.

In the hypertensive population, mean systolic BP was lower in patients under the care of more empathic physicians, but the difference did not reach statistical significance.

We found no differences between adequacy of BP management according to level of burnout. However, physicians with low burnout performed significantly better than their counterparts on several levels: they had lower systolic BP results for the overall population (p < 0.001) and the hypertensive population (p = 0.011) as well as a higher proportion of patients with a systolic BP <130 mmHg (p < 0.001).

### Burnout/empathy of nurses and BP management and control

The results for family nurses are shown in Tables 4 and 5. A majority of patients with good BP control were under the care of nurses with low burnout. The mean systolic BP for patients in general seen by nurses with low burnout was 127.6 mmHg (95% CI 127.5-127.8) compared with 128.9 mmHg (95% CI 127.5-130.4) for those seen by nurses with high burnout (p < 0.001). However, in the subgroup of hypertensive patients, mean systolic BP was significantly lower among those under the care of nurses with high burnout.

**Table 4:** Blood Pressure control and results of Nurse’s Empathy

Nurses		Low Empathy	Moderate Empathy	High Empathy	All	p
Patients under nurses’ care	n	52173	51298	49354	152825	
Patients with adequate blood pressure management^a^	n (%)	20197 (38.71%)	19883 (38.76%)	18581 (37.65%)	58661 (38.38%)	0.001^1^
	95% CI	[38.29% – 39.13%]	[38.34% – 39.18%]	[37.22% – 38.08%]	[38.14% – 38.63%]	
Systolic blood pressure in overall population	mean (SD)	128.09 (15)	127.59 (15.3)	128.29 (15.7)	127.98 (15.3)	<0.001^2^
	95% CI	[127.9 – 128.3]	[127.4 – 127.8]	[128.1 – 128.5]	[127.9 – 128.1]	
	median [IQR]	129 [120–138]	129 [118–137]	129 [119–138]	129 [119–137]	
Blood control levels in overall population						0.921^1^
<130 mmhg	n (%)	10172 (50.36%)	10257 (51.59%)	9463 (50.93%)	29892 (50.96%)	
	95% CI	[49.67% – 51.05%]	[50.89% – 52.28%]	[50.21% – 51.65%]	[50.55% – 51.36%]	
130 – 140 mmHg	n (%)	6167 (30.53%)	6039 (30.37%)	5437 (29.26%)	17643 (30.08%)	
	95% CI	[29.9% – 31.17%]	[29.73% – 31.01%]	[28.61% – 29.92%]	[29.71% – 30.45%]	
>140 mmHg	n (%)	3858 (19.1%)	3587 (18.04%)	3681 (19.81%)	11126 (18.97%)	
	95% CI	[18.56% – 19.64%]	[17.51% – 18.58%]	[19.24% – 20.38%]	[18.65% – 19.28%]	
Hypertensive Patients	n (%)	1464 (2.81%)	1535 (2.99%)	1518 (3.08%)	4517 (2.96%)	0.011^1^
	95% CI	[2.66% – 2.95%]	[2.84% – 3.14%]	[2.92% – 3.23%]	[2.87% – 3.04%]	
Systolic blood pressure in hypertensive population	mean (SD)	131.22 (14.3)	130.43 (15.5)	130.75 (15.9)	130.8 (15.2)	0.493^2^
	95% CI	[130.4 – 132.1]	[129.5 – 131.4]	[129.8 – 131.7]	[130.3 – 131.3]	
	median [IQR]	131.5 [121–140]	130 [120–139]	130 [121 – 139]	130 [121 – 139]	
Blood control levels in hypertensive population						0.086^1^
<130 mmHg	n (%)	417 (39.79%)	442 (41.82%)	446 (44.11%)	1305 (41.88%)	
	95% CI	[36.83% – 42.75%]	[38.84% – 44.79%]	[41.05% – 47.18%]	[40.15% – 43.61%]	
130 – 140 mmHg	n (%)	363 (34.64%)	379 (35.86%)	323 (31.95%)	1065 (34.18%)	
	95% CI	[31.76% – 37.52%]	[32.96% – 38.75%]	[29.07% – 34.82%]	[32.51% – 35.84%]	
>140 mmHg	n (%)	268 (25.57%)	236 (22.33%)	242 (23.94%)	746 (23.94%)	
	95% CI	[22.93% – 28.21%]	[19.82% – 24.84%]	[21.31% – 26.57%]	[22.44% – 25.44%]	

^a^ At least two correctly recorded readings taken on different days. ^1^: Chi-square linear-by-linear association Test ^2^: ANOVA test

**Table 5:** Blood Pressure control and results of Nurse’s Burnout

Nurses		Low Burnout	Medium Burnout	High Burnout	All	p
Patients undernurses’ care	n	96888	54441	1496	152825	
Patients with adequate blood pressure management^a^	n (%)	37500 (38.7%)	20593 (37.83%)	568 (37.97%)	58661 (38.38%)	0.001^1^
	95% CI	[38.4% – 39.01%]	[37.42% – 38.23%]	[35.51% – 40.43%]	[38.14% – 38.63%]	
Systolic blood pressure in overall population	mean (SD)	127.67 (15.1)	128.52 (15.7)	128.96 (17.8)	127.98 (15.3)	<0.001^2^
	95% CI	[127.5 – 127.8]	[128.3 – 128.7]	[127.5 - 130.4]	[127.9 - 128.1]	
	median [IQR]	129 [119–137]	130 [119–138]	130 [119–136]	129 [119–137]	
Blood control levels in overall population						<0.001^1^
<130 mmHg	n (%)	19354 (51.61%)	10258 (49.81%)	280 (49.3%)	29892 (50.96%)	
	95% CI	[51.1% – 52.12%]	[49.13% – 50.5%]	[45.18% – 53.41%]	[50.55% – 51.36%]	
130 – 140 mmHg	n (%)	11368 (30.31%)	6063 (29.44%)	212 (37.32%)	17643 (30.08%)	
	95% CI	[29.85% – 30.78%]	[28.82% – 30.06%]	[33.35% – 41.3%]	[29.71% – 30.45%]	
>140 mmHg	n (%)	6778 (18.07%)	4272 (20.74%)	76 (13.38%)	11126 (18.97%)	
	95% CI	[17.69% – 18.46%]	[20.19% – 21.3%]	[10.58% – 16.18%]	[18.65% – 19.28%]	
Hypertensive Patients	n (%)	2937 (3.03%)	1534 (2.82%)	46 (3.07%)	4517 (2.96%)	0.032^1^
	95% CI	[2.92% – 3.14%]	[2.68% – 2.96%]	[2.2% – 3.95%]	[2.87%3.04%]	
Systolic blood pressure in hypertensive population	mean (SD)	130.34 (15.3)	131.84 (15)	125.8 (17.6)	130.8 (15.2)	0.013^2^
	95% CI	[129.7 – 131]	[130.9 – 132.8]	[118.1 – 133.5]	[130.3 – 131.3]	
	median [IQR]	130 [120–138.5]	132 [122–140]	126 [112.5 – 141]	130 [121–139]	
Blood control levels in hypertensive population						0.021^1^
<130 mmHg	n (%)	886 (42.6%)	408 (40.16%)	11 (55%)	1305 (41.88%)	
	95% CI	[40.47% – 44.72%]	[37.14% – 43.17%]	[33.2% – 76.8%]	[40.15% – 43.61%]	
130 – 140 mmHg	n (%)	733 (35.24%)	328 (32.28%)	4 (20%)	1065 (34.18%)	
	95% CI	[33.19% – 37.29%]	[29.41% – 35.16%]	[2.47% – 37.53%]	[32.51% – 35.84%]	
>140 mmHg	n (%)	461 (22.16%)	280 (27.56%)	5 (25%)	746 (23.94%)	
	95% CI	[20.38% – 23.95%]	[24.81% - 30.31%]	[6.02% - 43.98%]	[22.44% - 25.44%]	

^a^. At least two correctly recorded readings taken on different days. ^1^: Chi-square linear-by-linear association Test ^2^: ANOVA test

Significant differences were found for mean systolic BP for the overall population according to nurses’ level of empathy, but in the hypertensive group, mean systolic BP was significantly lower in patients monitored by more empathic nurses (p = 0.01).

While nurses with high empathy performed significantly fewer BP checks than those with low empathy (p = 0.01), like physicians, those with low burnout performed more checks (p = 0.01).

## Discussion

Mean systolic BP was significantly lower in patients under the care of physicians with low burnout for both the overall population (p = 0.001) and patients with hypertension (p = 0.011). In other words, burnout among family physicians was significantly associated with BP levels. In the case of empathy, we found evidence of better BP control for hypertensive patients under the care of more empathic physicians. In the general population, BP levels were lower in patients under the care of less empathic professionals but the association was not statistically significant. To our knowledge, this is the first study to investigate how levels of burnout and empathy among primary care providers in our health care district might affect clinical outcomes.

Although PRESCAP 2010, a cross-sectional multicenter study conducted in Spain, investigated how primary care physicians manage hypertension in routine practice, it did not consider factors related to either empathy or burnout [28]. In our study population, high empathy was significantly associated with low burnout, supporting the theory that actions designed to improve communication skills and empathic tendencies among health care professionals could help to mitigate burnout.

Although we found a positive association between high empathy/low burnout and better BP control and management, there are no similar studies with which to compare our results, apart from that by Hojat et al.,[14] which detected a significant association between physician empathy and metabolic control in diabetes patients. Indeed, a recent systematic review and meta-analysis of the influence of the patient-clinician relationship on health care outcomes found that very few studies had detected a significant association between empathy and clinical benefits [29].

Empathy, by contrast, has been shown to have a positive impact on the doctor-patient relationship in terms of immediate outcomes, such as greater patient satisfaction [30] and treatment adherence [31]. Adherence to treatment and physician recommendations is a key component of good BP control, particularly in the early stages of management, where lifestyle modifications can have an impact. It has been proposed that empathically engaged physicians communicate more openly with their patients, fostering a climate of conversation in which patients are encouraged to talk about their symptoms and their fears, and in which physicians explain the nature of patients’ disease and the different aspects of treatment [32]. In 2007, a German team found physician empathy to be associated with improvements in several long-term outcomes reported by patients with cancer, including depression and pain perception [33].

In our series, family physicians with high empathy scores were significantly more likely to achieve good BP control, as the patients under their care, including those with hypertension, had a lower mean systolic BP than those under the care of physicians with lower scores on the JSPE. They also had a higher proportion of patients with a systolic BP of <140 mmHg. Although the above differences have many potential explanations, it is possible that patients seen by more empathic physicians are more inclined to follow treatment and lifestyle advice.

Our results for burnout were similar in that physicians with low burnout had significantly better BP control results than those with high burnout (more patients with a systolic BP <140 mmHg and a significantly lower mean systolic BP in both the overall and hypertensive populations).

Patients under the care of family nurses with high empathy scores also had better BP control rates than those seen by nurses with low empathy. However, contrasting with the situation observed for nurses with low burnout, more empathically engaged nurses performed fewer BP tests. We observed both a lower mean systolic BP and a higher number of patients with a systolic BP <140 in both the overall and hypertensive populations, suggesting perhaps that empathic nurses, rather than simply measuring blood pressure, take an active role in encouraging actions than can help to reduce hypertension. While we also observed better BP results in the overall population under the care of nurses with low burnout, we were surprised to find that the opposite was true for the hypertensive population.

Using indicators such as good verbal and non-verbal communication and time spent with patients, several studies have suggested that empathic engagement by physicians can lead to increased patient satisfaction [34, 35] and treatment adherence. Our results suggest that this might also be true in the primary care setting, as better control of hypertension, a condition in which both pharmacologic and non-pharmacologic treatments are important, was achieved by more empathic primary care providers. In future studies, it would be interesting to analyze treatment adherence among hypertensive patients according to levels of physician empathy or burnout.

Our study has both strengths and limitations. Strengths include the high response rate and large sample size, as we analyzed BP data for over 300,000 patients under the care of 60% of all primary care providers in our health district. One of the main limitations of our study is that although we observed statistically significant differences between levels of BP control according to levels of burnout and empathy, we cannot know whether these differences resulted in actual clinical improvements. Because our sample size was so large, it is possible that some associations might have had statistical but not clinical significance. The possibility of a white-coat effect should also be considered, although the effect would have been similar for patients with or without hypertension.

Another limitation of our study is that the physician and nurse burnout and empathy levels were based on questionnaire responses, and there is an obvious risk that the respondents may sometimes have answered what they thought was “expected”, rather than what they truly believed. While this risk is greater in the case of socially sensitive subjects such as empathy and burnout, we believe it was minimized by the use of two widely used and validated questionnaires: the MBI and the JSPE. The interpretation of our findings is also limited by a lack of comparative data.

We believe that the limitations associated with the empathy and burnout questionnaires are acceptable as these tests have been amply validated and widely used to analyze empathy and burnout in other studies. In our study, we first analyzed empathy and burnout levels and then looked at BP results for patients under their care. We could not have done this the opposite way around as this would have been a breach of confidentiality, although such an approach would certainly have improved the power of the study.

Finally, we did not control for the multiple factors that could influence BP control, such as treatment adherence, age, and concomitant disease. Moreover, the BP measurement heterogeneity can be considered as a limitation because we cannot assure that all the professionals measure BP in the same way (, patient standing, sitting, laying, with or without a rest of different durations).

Our primary aim was to investigate a possible association between levels of empathy and burnout among family physicians and nurses and different markers of BP management. We did not study other potentially spurious variables. We are well aware that multiple factors can influence empathy, burnout, and BP control, such as patient age, number of years working at the same centers, organization of the medical team, etc. Information on these factors, however, was not available in the database, so we simply analyzed the association, without controlling for other factors. It would also be interesting in future studies to investigate different factors that can influence burnout and empathy, such as working environment and personal, family, and social factors.

## Conclusions

Despite the small effect size, we found that high empathy and low burnout influence BP control, and therefore may result in improved clinical outcomes in primary health care settings. However, more studies are required to confirm clinical differences depending on professionals’ characteristics.

We believe that patients have a key role in improving high BP, a disease known to have multiple causes. We also believe that health care professionals can play a crucial role in actively engaging patients in their own care, and work hand in hand with them to attain health goals.

This is preliminary and exploratory study that determined the distribution of burnout and level of empathy in both nurses and physicians in our region, finding potentially important relationships between empathy and burnout and some intriguing statistical association with important patient level variables for which there is evidence that burnout and empathy of clinicians could be affecting clinically important results.

### Practice Implications

The promotion of skills that improve empathy and reduce burnout among health care professionals could benefit both care providers and patients. Our finding that patients under the care of more empathic and less burned-out physicians had better BP control highlights the importance of actions designed to increase empathy and reduce burnout among health care professionals and primary care gatekeepers in particular. Our findings should also encourage health care providers to reflect on how their levels of burnout and empathic engagement might be affecting their daily practice.

Finally, our results should pave the way for new lines of research on how health care providers’ empathic tendencies and communication skills can be improved with the ultimate goal of improving clinical outcomes, and can lead to new projects using methods that strengthen the likelihood of observational studies revealing actual “truths” about practice.

## Abbreviations

BP: Blood pressure
MBI: Maslach Burnout Inventory
JSPE: Jefferson Scale of Physician Empathy
